# Recent Progress in Droplet Structure Machining for Advanced Optics

**DOI:** 10.3390/mi15030337

**Published:** 2024-02-28

**Authors:** Jin-Kun Guo, W.D.N. Sandaruwan, Jinwei Li, Jinzhong Ling, Ying Yuan, Xin Liu, Qiang Li, Xiaorui Wang

**Affiliations:** School of Optoelectronic Engineering, Xidian University, Xi’an 710071, China

**Keywords:** droplet, liquid crystal, soft matter, microfluidics, laser micro–nano-machining, 3D printing, emulsions, laser injection, microfabrication, lab on a chip

## Abstract

The development of optical and photonic applications using soft-matter droplets holds great scientific and application importance. The machining of droplet structures is expected to drive breakthroughs in advancing frontier applications. This review highlights recent advancements in micro–nanofabrication techniques for soft-matter droplets, encompassing microfluidics, laser injection, and microfluidic 3D printing. The principles, advantages, and weaknesses of these technologies are thoroughly discussed. The review introduces the utilization of a phase separation strategy in microfluidics to assemble complex emulsion droplets and control droplet geometries by adjusting interfacial tension. Additionally, laser injection can take full advantage of the self-assembly properties of soft matter to control the spontaneous organization of internal substructures within droplets, thus providing the possibility of high-precision customized assembly of droplets. Microfluidic 3D printing demonstrates a 3D printing-based method for machining droplet structures. Its programmable nature holds promise for developing device-level applications utilizing droplet arrays. Finally, the review presents novel applications of soft-matter droplets in optics and photonics. The integration of processing concepts from microfluidics, laser micro–nano-machining, and 3D printing into droplet processing, combined with the self-assembly properties of soft materials, may offer novel opportunities for processing and application development.

## 1. Introduction

The micro- and nano-machining techniques applied to solid materials have yielded remarkable success in the semiconductor industry by integrating complex functionalities into microscale devices, thus spearheading the modern electronics revolution [1]. Extending similar miniaturization strategies to process and assemble soft matter for creating multileveled functional structures over various length scales presents significant scientific and practical potential [2,3,4]. Soft matter, including liquid crystals (LC), colloids, polymers, and biological substances, exhibits widespread influence across nature, living organisms, daily life, and industry [2,5,6]. The biomimetic properties, responsiveness to stimuli, and efficacy in controlled release and sensing make soft matter extensively applicable in biology and chemistry [7,8,9,10,11,12,13,14,15,16,17,18,19]. Additionally, soft matter offers distinct advantages over solids in optical and photonic applications owing to its inherent adaptability, tunability, and seamless integration capabilities [3,20,21,22,23,24,25]. These unique properties open avenues for pioneering optical designs, adaptive systems, and versatile devices capable of dynamic responses to changing environmental or operational conditions.

Soft matter in mesoscale is profoundly influenced by surface tension, resulting in the spontaneous formation of curved geometries such as spheres or domes when dispersed in other immiscible liquids or air [4]. The curved confinement that encloses soft matter into separate functional units facilitates miniaturization but also poses a barrier to its micro- and nano-machining. However, the three-dimensional (3D) microstructures and the distribution of material composition within the closed confinement are crucial to its optical behavior, playing a pivotal role in the development of applications in optics and photonics. These factors govern the interaction of light, influencing properties such as refraction, reflection, scattering, and the ability to function as optical elements [26]. Therefore, precise micro- and nanofabrication of soft-matter droplets is essential to advance their development and explore their potential.

This review aims to provide an overview of the recent advancement in the machining of droplet structures of soft matter and their emerging applications in optics and photonics. Firstly, we present three types of typical droplet structures and three techniques for droplet structure machining, including the principles and characteristics. Microfluidics is the most successful machining technique in the field, being both widely adopted and discussed. In particular, we introduce a strategy for introducing phase separation in microfluidics to build complex droplet structures. Additionally, we focus on two new technologies for assembling droplet structures from the bottom–up approach, namely laser injection and microfluidic 3D printing. Secondly, we introduce applications of soft-matter microdroplets as independent functional units in optics and photonics, including droplet lasers, waveguides, lenses, display and information tags, and others. Finally, we address the challenges and opportunities for future applications of droplet-based microsystems in the realm of optics and photonics from the perspective of droplet structure machining.

## 2. Typical Droplet Structure of Soft Matter

**Figure 1:**
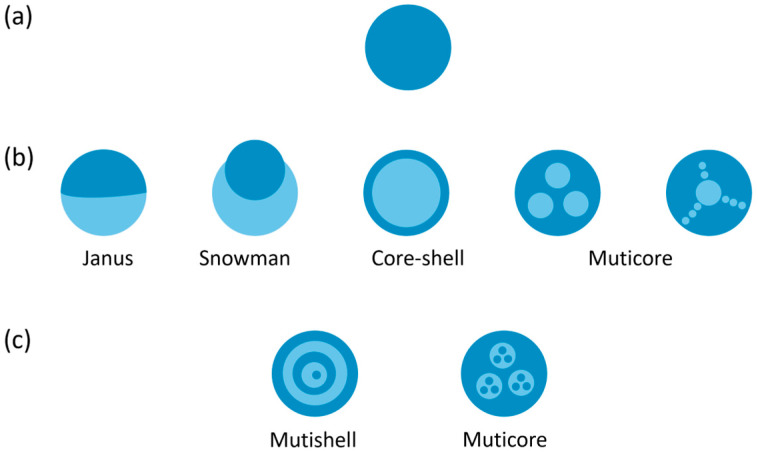
Distinct configuration of single emulsion droplet (**a**), double emulsion droplet (**b**), and multiple emulsion droplet (**c**).

An emulsion droplet is a non-homogeneous liquid structure in soft matter, where one liquid is dispersed in another immiscible liquid in the form of tiny droplets [10]. As illustrated in Figure 1, droplets can be categorized based on their hierarchical structure into single emulsions, double emulsions, and multiple emulsions. The simplest form is a single emulsion droplet, for example, water-in-oil or oil-in-water. In cases where more than two phases are involved in emulsion, the dispersed droplets may contain multiple compartments. When two compartments are brought into contact, they can form structures such as Janus, Snowman, and core–shell, determined by the balance of interfacial tensions [27]. The number of inner compartments can exceed one, resulting in a multicore structure. These compartments can either be freely suspended or self-assemble into ordered substructures with the help of their surrounding liquid (Figure 1b) [10,28]. Multiple emulsions in soft matter are complex polydisperse systems where both oil in water and water in oil emulsion exist simultaneously (Figure 1c).

## 3. Strategies for Crafting Soft-Matter Droplet Structures

### 3.1. Microfluidics

**Figure 2:**
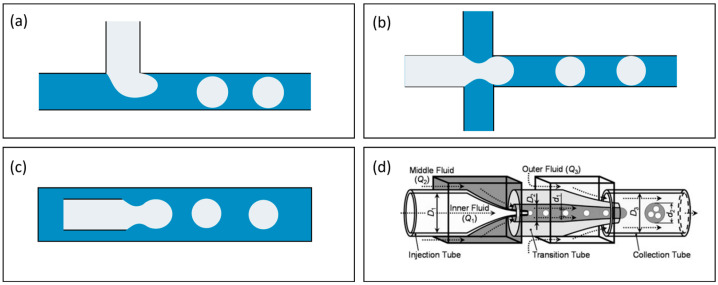
Typical microfluidic channel geometry for droplet generation: (**a**) T-junction, (**b**) flow-focusing, and (**c**) co-flowing; (**d**) schematics for multistep emulsification to produce complex emulsion droplets by cascading more channels. Reprinted from Ref. [33], with permission from Wiley.

Microfluidics is the most widely applied technique in droplet structure machining [4,11,13,29,30,31,32]. This technique employs microfluidic chips with intricately designed microchannels or chambers to control the convective flow of immiscible liquids, leading to the controlled formation of microdroplets under shear or squeezing forces (Figure 2a–c). The process can extend further to two-step or even multistep assembly to craft the 3D architecture of droplets as required (Figure 2d) [33,34]. Microfluidic devices come in various geometries, including T-junction, flow-focusing, and co-flowing, each offering unique advantages. Numerous comprehensive papers have summarized the technical principles and characteristics of these microfluidic chips [11,13,35,36]. Over the years, microfluidics has advanced to achieve controlled design and generation of droplet size, shape, structures, and function, showcasing significant advancements in the field.

The precision of microfluidics machining for droplet structures relies on the capability to accurately control fluids, a feature dictated by the microchannel structure. The fabrication of microfluidic devices, whether using polymers or glass capillaries, with sophisticated channels entails demanding process conditions and precision instruments, such as soft photolithography, plasma cleaners, micropipette pullers, and microforges [11,37,38]. Moreover, considerable expertise is necessary for the proper surface treatment of different channel segments, precise alignment of channel parts, and reliable assembly of channel architectures. Therefore, the availability of this technology is limited by its cost and the need for professional microfabrication technologies.

**Figure 3:**
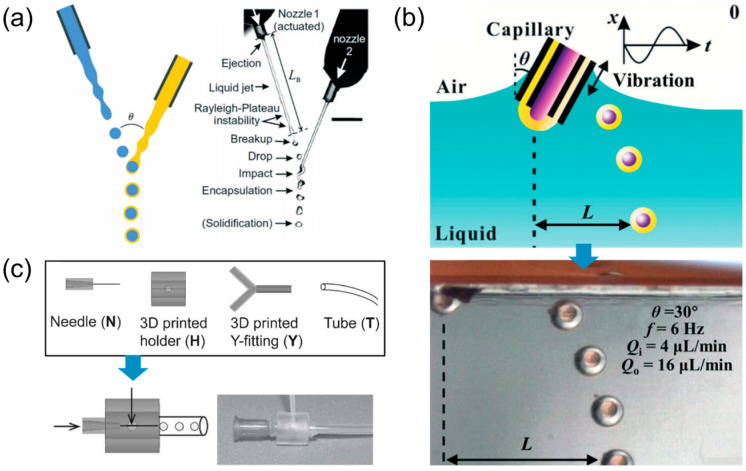
Schematic drawing and device image for droplet generators. (**a**) in-air microfluidic method; (**b**) coaxial oblique interface shearing method; (**c**) microfluidic chip with 3D printed fittings. Reprinted from Ref. [39] under the terms of Creative Commons attribution noncommercial license 4.0 (CC BY-NC). Adapted from Ref. [40], with permission from Royal Society of Chemistry.

In the field of microfluidics, extensive studies devote efforts to the mass production of well-defined droplets, eliminating the necessity for complex microfluidic chips and specialized expertise. One notable approach involves an in-air chip-free method that employs two nozzles to discharge liquid jets (Figure 3a) [39]. This method induces droplet formation by allowing the spontaneous enwrapping of various liquids. Similarly, a technique utilizes vibrations applied to a coaxial needle to generate droplets, inducing interfacial shear without the requirement for chips (Figure 3b) [40,41]. Additionally, innovative approaches have been developed to streamline the fabrication of a microfluidic flow-focusing device. This is achieved by leveraging 3D-printed fittings and fluidic modules, allowing for plug-and-play functionality (Figure 3c) [10,31,42,43,44,45,46].

**Figure 4:**
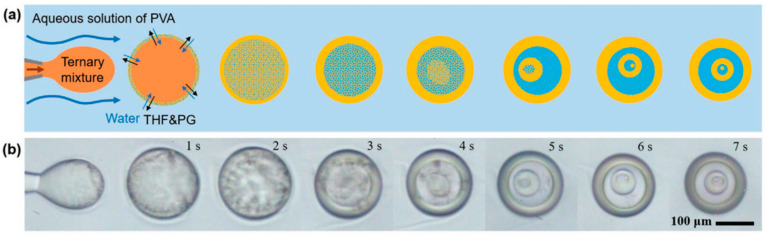
Schematic (**a**) and microscopic images (**b**) showcasing the fabrication of multiple emulsion droplets with intricate multishells via phase separation. Adapted from Ref. [48] with permission from Wiley.

Despite its development, microfluidics encounters limitations in processing soft matter due to its direct contact and top–down manner. The distinctive physical properties of soft matter, such as fluidity, weak shear resistance, surface tension, and diffusion, pose significant constraints on microfluidic chip design [47]. Consequently, this technology is primarily capable of producing relatively simple structures, such as spheres of one fluid embedded in a sphere of another fluid [4]. Building upon this foundation, in addition to multistep emulsification by cascading additional microfluidic channels, complex droplet structures can also be assembled by leveraging the phase separation. Through controlled liquid-liquid phase separation in the microfluidic channel, the produced droplet can transform into onion-like structures with multiple shells (Figure 4) [48]. The number of droplet shells, which can reach up to five, is adjustable by manipulating the initial composition of the ternary mixture, determining the successive steps of phase separation.

**Figure 5:**
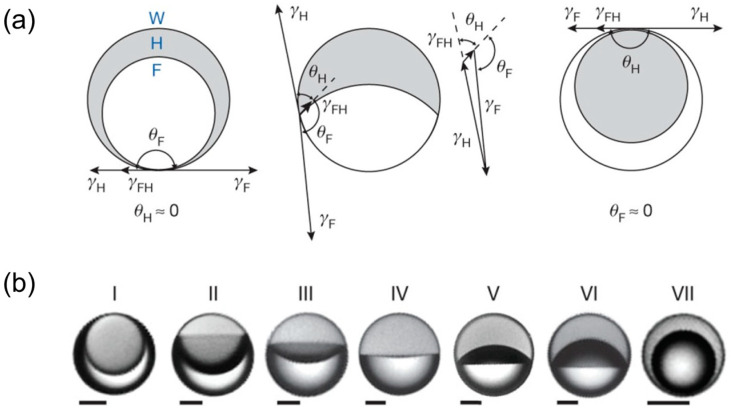
(**a**) Sketch of the effect of interfacial tensions on the configuration of a complex droplet. (**b**) Droplets continuously reconfigure from I to VII in response to variation in the concentration ratio of surfactant mixture. Adapted from Ref. [49] with permission from Springer Nature.

In addition, another novel method has been developed to dynamically reconfigure the geometry of a stabilized droplet by adjusting interfacial tensions [27,49,50]. The processing involves two steps. In the first step, two oil-like liquids (hydrocarbon and fluorocarbon), which can only mix above a certain temperature, are mixed together and then dispersed in water to form droplets. Upon cooling, two liquids inside each droplet separate into two distinct phases due to phase separation, forming a double emulsion droplet. In the second step, a specifically designed surfactant mixture is utilized to alter the interfacial tension between the two oils and the water. As a result, the droplet geometries can be dynamically alternated between encapsulated core–shell, and Janus configurations by the balance of these three interfacial tensions (Figure 5). These droplet configurations are characterized by two contact angles, *θ_H_* between the H-W and F-H interfaces and *θ_F_* between the F-W and F-H interfaces (Equation (1)) [27,49].

(1) $$\begin{array}{c} cos\left(\theta_{H}\right)=\frac{\gamma_{F}^{2}\;-\;\gamma_{H}^{2}\;-\;\gamma_{FH}^{2}}{2\gamma_{FH}\gamma_{H}},\\ cos\left(\theta_{F}\right)=\frac{\gamma_{H}^{2}\;-\;\gamma_{F}^{2}\;-\;\gamma_{FH}^{2}}{2\gamma_{FH}\gamma_{F}}, \end{array}$$

where *γ* is the interfacial tension with different subscripts to represent three interfaces. This method can be further expanded to tailor droplet geometries by adding different chemicals or exposing them to light or to different acidity levels.

Finally, microfluidics faces challenges in precisely controlling the internal architectures of droplets, particularly concerning the spatial arrangement and distribution of the plural substructures. It is important to note that soft matter exhibits both the fluidity of a liquid and the long-range ordering of a crystal, allowing its molecular orientational field to guide the arrangement of plural substructures through self-assembly [51]. The fusion of microfluidics and the self-assembly of soft matter serves as an extraordinary path of combining top–down and bottom–up structuring approaches [4]. However, during the machining of soft matter using microfluidics, its molecules are randomly arranged. The establishment of its orientational field typically takes hours or even days, and before that, self-assembly is not working [52]. This limitation restricts the full potential of the approach and the development of optical and photonic applications based on soft-matter droplets.

### 3.2. Laser Injection

**Figure 6:**
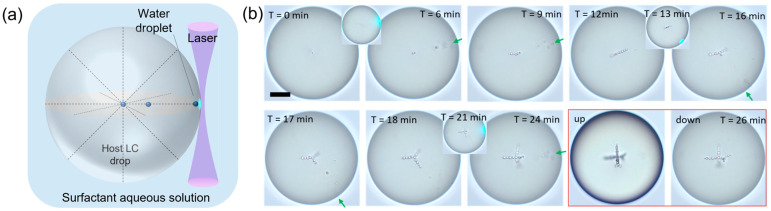
(**a**) Schematic drawing for laser injection; (**b**) Tailored injection and guided assembly of water droplet in a host LC droplet. This method is capable of fabricating droplets with out-of-equilibrium internal architectures. Adapted from Ref. [28] under the terms of Creative Commons CC BY license.

Laser injection technology is an emerging technique for customizing the internal architecture of a droplet in a bottom–up manner [28,52,53,54,55]. The microfabrication is initiated by the injection of nanoscale water droplets into a pre-selected site on the surface of a stabilized LC (one type of soft matter) droplet through laser irradiation; no syringe or nozzle is required (Figure 6a). Leveraging the elasticity of the LC, the injected water solution spontaneously forms into monodispersed droplet cores and undergoes a series of self-assembly processes within the host LC droplet [28,56]. This intricate sequence involves growth, movement, and interaction with the background orientational field of the LC, resulting in the formation of ordered plural sub-structures. The size of injected water droplets is determined by the anchoring extrapolation length ξ = K/W, typically on the order of a micrometer [57,58]. It can be further adjusted by altering the elastic constant of the LC (K) and the anchoring coefficient (W) by varying the temperature and surfactant concentration, respectively. Ultimately, customized fabrication of the 3D architecture of an LC droplet is achieved by precisely adjusting the laser beam and irradiation site, thereby controlling the self-assembly dynamics of injected water cores (Figure 6b). 

The controllable assembly of droplets (or colloids) on a large scale in open spaces using LC has been extensively reported [57,59,60]. The key lies in intervening in their self-assembly process by transporting droplets to the specified location in a stable orientational field of LC. Fortunately, the laser injection technology provides an available tool to overcome the surface confinement and loading water droplet cores into the LC droplet through a pre-selected site. Therefore, it achieves complete intervention in the self-assembly dynamics of the injected droplets by means of injecting them into a host LC droplet with a stable orientational field [28,55,61]. This eliminates the barrier to machining the internal architecture of a droplet in a bottom–up manner based on self-assembly, resulting in high machining accuracy.

**Figure 7:**
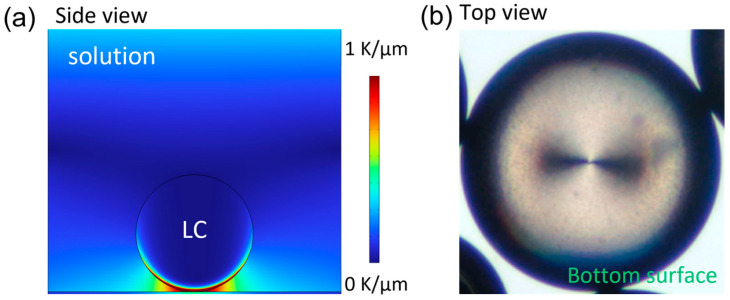
The injection site is determined by temperature distribution. (**a**) The induced temperature gradient based on the FEM simulation. (**b**) Injection site covers the bottom surface of the droplet. Adapted from Ref. [55] with permission from American Chemical Society.

Laser injection primarily relies on the interaction between light and matter. Specifically, a Gaussian laser beam functions as a localized heat source, inducing instabilities at the water-LC interface and resulting in the injection of water across the interface [28]. A similar injection process can also be achieved through direct temperature control [55]. Although the concrete mechanism requires further quantitative studies, it has been confirmed that the interface injection is primarily driven by the thermally induced Marangoni effect. As the injection site on the interface is determined by the temperature distribution, and even the total volume of the injected water solution can be precisely controlled by adjusting the induced Marangoni force (*Fσ*, Equation (2)) through the applied temperature difference and cooling rate (Figure 7) [55,62,63].

(2) $$F_{\sigma }=\frac{8\pi R^{2}}{3}\frac{d\sigma }{dT}\left|\frac{dT}{dr}\right|,$$

where *R* is the radius of the LC droplets, *dσ*/*dT* is the thermal dependence of the surface tension at the LC–water interface, and *dT*/*dr* is the temperature gradient over the LC–water interface. It has been demonstrated that the key to a successful injection lies in generating sufficient Marangoni force, elucidating the minimum laser energy requirement for laser injection. It is estimated that a typical 455 nm laser exposure, with a 10 μm beam and 4 mJ (0.2 mW × 20 s) energy, can raise the temperature by 6 °C, induce a surface tension gradient of 8 N/m, and generate a Marangoni force with a magnitude of 0.2 μN. This force is one or two orders of magnitude higher than that achieved with a laser tweezer [64,65]. 

**Figure 8:**
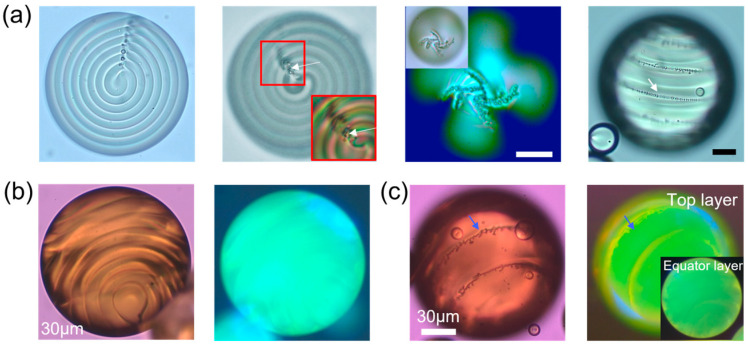
(**a**) Laser injection and controlled self-assembly processes yield droplet structures of unprecedented complexity. Adapted from Ref. [54] under the terms of Creative Commons attribution noncommercial license 4.0. (**b**,**c**) Laser machining of optical microstructures in 3D, showcasing a cholesteric liquid crystal (LC) droplet emitting distinct fluorescence lights from its body and substructures under UV illumination (**c**). Adapted from Ref. [52] with permission from © Optical Society of America.

Moreover, laser injection technology has the capability to load any number of water droplets into pre-selected locations on the LC droplet surface, overcoming the limitation of its spherical confinement. This capability enables the machining of droplet inner architecture with unprecedented complexity alongside customized assemblies (Figure 8). The sequentially injected water droplets spontaneously drifted toward areas with topological defects and self-assembled along its geometry or local director field of LC into a predefined shape. Within cholesteric LC (CLC) droplets with different topological structures, guest droplets self-assembled near areas with defect points as twisting radial chains and quill-like assembly structures and along defect lines as discrete beads, helical threads, and surface rings, respectively (Figure 8a). Additionally, by doping functional materials into the surrounding water solution, laser injection is also capable of controlling the chemical composition within each droplet core (Figure 8b,c) [52,66,67].

**Figure 9:**
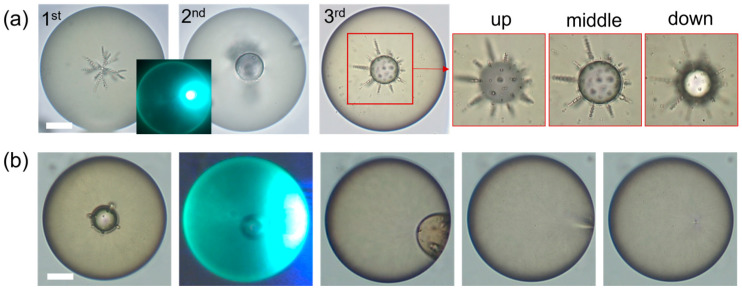
(**a**) Laser-induced coalescence and self-assembly of guest droplets enable reconfigurable assembly architecture. Three magnified images correspond to different microscope focal planes. (**b**) Droplet release via high-power laser exposure (60 mW, 20 s) melting the LC (clear point at 57 °C). Scale bars: 10 µm. Adapted from Ref. [28] under the terms of Creative Commons CC BY license.

Furthermore, laser injection technology provides a versatile light toolbox for droplet machining, including laser injection, laser-induced coalescence, and laser-induced reconfiguration. Beyond injection, the technique involves rapidly altering the local temperature at specific points using lasers with different powers (achieving a rapid change in the K value). This process can induce secondary merging of injected water droplets or assist them in escaping from the host LC droplets (Figure 9). The capability to adjust and reconfigure the internal architecture of a droplet is facilitated by this process. Moreover, its non-contact and in situ machining manner makes it possible to process droplets of different materials and sizes without limitations. Consequently, it provides a high degree of freedom in machining droplet structures. 

Despite its considerable potential, laser injection requires many improvements to reach a level of maturity and reliability. First, there is a need for improved machining efficiency. In the current stage, with continuous laser exposure, it takes 20 s for each injection, presenting a significant gap compared to the highly efficient micro–nano-machining of solid materials achievable with pulsed lasers. Second, extended periods of laser processing can result in heat deposition, leading to a degradation in machining precision. A meticulous analysis of the temperature field distribution and evolution under the photothermal effect becomes imperative to achieve precise control and enhance the machining capabilities of this technology. Third, a quantitative exploration of the physical mechanisms governing laser-induced interfacial injection is indispensable for elucidating the core of this process. Such an investigation is pivotal for propelling the technology toward becoming a generalized liquid/liquid injection processing method.

### 3.3. Microfluidic 3D Printing

Three-dimensional printing, also known as additive manufacturing, encompasses methodologies employed to construct three-dimensional objects based on computer-aided designs. Most printing technologies fall into two categories: light-based or ink-based methods, determined by their use of either curing light-sensitive materials through exposure to light or directly depositing inks with specific viscoelastic properties [2]. These techniques provide significant capability to quickly turn computer-aided design into 3D items with elaborate architectures as required. In addition, their bottom–up feature allows for printing devices on nonplanar surfaces and facilitates the integration of multipart systems in spatial dimensions [68,69,70]. These aspects hold great importance in the field of photonics and optical applications. 

For the 3D printing of soft matter, typical light-based methods include two-photon polymerization, digital light processing, stereolithography, and continuous liquid interface production [2,71]. These technologies offer high resolution, accuracy, geometric complexity, and fabrication speed [68]. However, they encounter limitations in the selection of processable materials and challenges in implementing multimaterial 3D printing [2,6,68]. In contrast, ink-based methods such as liquid-in-liquid 3D printing (LL3DP) and layer-by-layer patterning hold promise in addressing the aforementioned challenges associated with printing soft matter. Specifically, the LL3DP makes it possible to shape soft matters into freeform complex 3D designs but with relatively low resolution and speed [6].

**Figure 10:**
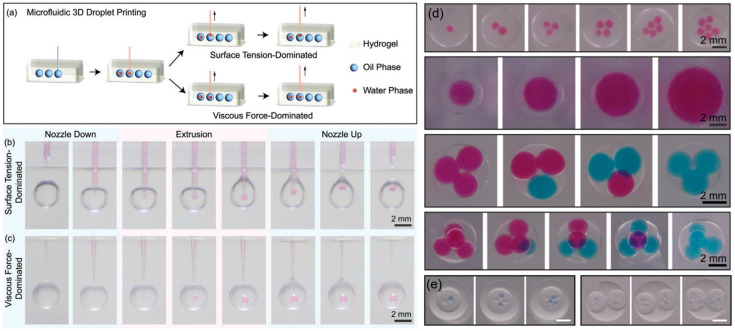
Microfluidic 3D droplet printing. (**a**) Schematics illustrating surface tension and viscous force-dominated droplet printing. Snapshots depicting the processes of (**b**) surface tension-dominated and (**c**) viscous force-dominated droplet printing. Double emulsion droplets (**d**) and Triple emulsion droplets (**e**) produced using this method. Adapted from Ref. [72] with permission from Wiley.

The utilization of 3D printing extends to droplet machining, as demonstrated by microfluidic 3D printing technology operating on the principles of LL3DP [72,73]. In this method, water solution is injected into a closed oil droplet to form droplet cores using a nozzle, resembling the process of a dragonfly laying eggs (Figure 10a). The detachment of droplet cores from the nozzle is intricately influenced by surface tension and viscous forces. Notably, this technique showcases the fascinating capability to construct droplets with tunable core numbers, core sizes, and core compositions (Figure 10b). Moreover, it has the capability to fabricate multiple emulsion droplets (Figure 10c). Despite limitations imposed by the 3D printer’s resolution and nozzle diameter, this approach holds significant potential for droplet structure design and engineering.

## 4. Applications of Droplet Microsystems in Optics and Photonics

### 4.1. Droplet Lasers 

**Figure 11:**
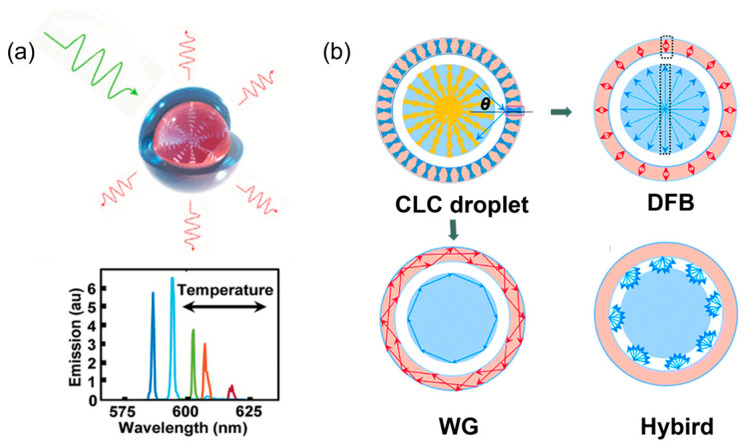
(**a**) Schematics for dye-doped CLC microdroplet laser with internal radial helix director profile. Temperature tunable lasing, indicated by changes in color, occurs via changes in LC molecular helical pitch. (**b**) Cross profile of a CLC triple emulsion droplet. Ray optic schematics for optical resonances: shell and core DFB resonances, TIR-based WG resonances in shell and core, hybrid resonance with optical reflections on Bragg shell. Adapted from Refs. [75,76] with permission from American Chemical Society and Royal Society of Chemistry, respectively.

The microlaser based on droplets stands out as a promising technology, with its distinctive feature of flexibility and compactness [4,48,74]. The spherical shape of the droplet makes it an ideal candidate to act as a resonance cavity. By incorporating the gain media into the cavity, droplets enable controllable lasering under pump radiation [26]. When employing LC as the active medium, the system acquires dynamic and tunable optical properties, thereby enhancing the versatility and adaptability of the microlaser (Figure 11a) [75]. LC molecules in cholesteric phase are noteworthy for their spontaneously self-assemble into periodic helical structures exhibiting a photonic bandgap [76].

Since the first discovery of CLC droplet lasers by Musevic et al., there has been substantial progress in developing this system as a tunable wavelength lasing source [77]. The primary focus has been on exploring new cavity design and fabrication approaches to integrate one or more gain media with geometry-dependent modes (Figure 11b). Three types of laser resonators can be obtained by altering the droplet structure, such as droplet, core–shell, and multishells, as well as by incorporating gain dyes and CLCs in different cavities. First, once confined CLC within a sphere or a shell with parallel anchoring, LC molecules arrange themselves into a Bragg-onion structure, resulting in laser emitting in band-edge mode (also called distributed feedback or DFB mode) [76]. Second, the spherical cavity contributes to forming Whispery Gallery (WG)-mode lasing based on the light total internal reflections on the boundary of the core and shell [76,78]. Third, once CLC is confined in the outer shell and fulfills certain conditions, lasing in distributed Bragg reflection (DBR) mode can be achieved [74]. By controlling the spatial coupling between the pump beam and droplet, different modes or wavelengths can be selectively excited [48]. 

### 4.2. Waveguide 

**Figure 12:**
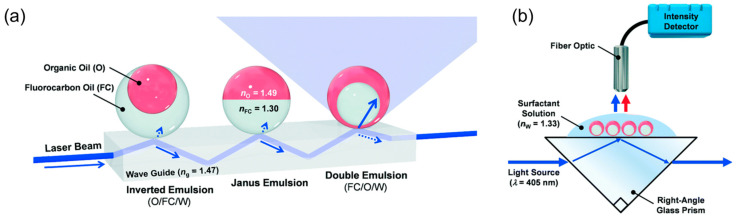
(**a**) Conceptual sketch of the modular waveguide comprising dynamic complex emulsions in three different geometries. Beginning on the left, total internal reflection (TIR) is observed for inverted and Janus emulsions. Upon transition to a double emulsion with the higher index organic phase on the outside, the laser light is out-coupled from the waveguide, thereby increasing light intensity measured above the waveguide. (**b**) Waveguide-based sensing device and optical read-out of changes in droplet geometry. Adapted with from ref. [81] under the terms of Creative Commons attribution 3.0 unported license.

An optical waveguide is a physical structure designed to confine light within its boundaries, ensuring efficient transmission through mechanisms like total internal reflection and evanescent coupling. This design offers precise control over the trajectory of light, guiding it along specific paths. Notably, droplets can serve as transduction units for light, as demonstrated by Timothy M. Swager et al., who developed optical waveguide-based sensors utilizing complex emulsion droplets [79,80,81]. These emulsion droplets consist of two immiscible liquids with distinct refractive indices, serving as a transduction unit. A unique feature of these emulsion droplets is their ability to undergo changes in geometry or orientation triggered by specific chemical or biological stimuli. This dynamic property allows for the tunability of the refractive index in emulsion droplets, facilitating the manipulation of light out-coupling (Figure 12a). 

As illustrated in Figure 12b, light propagating through a glass waveguide will undergo total internal reflection at the interface when in contact with low refractive index media. However, proximity to a higher refractive index medium can lead to light out-coupling from the waveguide. Researchers can detect and quantify stimuli components by analyzing changes in transmission intensity or scattered light patterns caused by structural changes in droplet droplets. The precise control of emulsion droplets over translation, rotation, and assembly has been extensively demonstrated by using magnetic nanoparticles. These could further enhance the behavior of emulsion droplets as compact, near-real-time waveguides. The dynamic nature of droplets allows for the creation of reconfigurable waveguides, offering adaptability in routing light signals. 

### 4.3. Microlens

**Figure 13:**
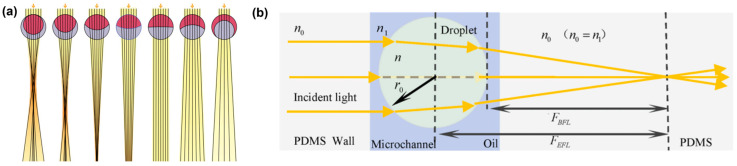
(**a**) Double emulsion droplets with distinct geometries can focus or diverge the light rays. Adapted from Ref. [50] under the terms of Creative Commons CC BY license. (**b**) Light-focusing diagram of a single-droplet microlens within optofluidic microlens arrays. Adapted from Ref. [83] with permission from American Chemical Society.

Droplets, acting as microscale lenses, harness the optical phenomenon of focusing light through their curved surfaces [82]. This natural effect is akin to the magnification observed when viewing objects through water droplets or the controlled focusing of light within microfluidic systems. A noteworthy application of droplet-based optics is found in liquid microlenses, offering tunable focal lengths achieved through the controlled deformation of liquid droplets. These unique lenses are crafted by combining two immiscible liquids, hydrocarbon, and fluorocarbon, creating bi-phase emulsion droplets. The capability to alter the droplet’s geometry allows for the regulation of whether it concentrates or scatters incoming light, providing versatile optical functionalities (Figure 13a) [50]. Depending on the setup, the lenses can either provide focused images of objects, as regular lenses do, or magnified virtual images. The geometry adjustment of the droplet can be achieved through external stimuli such as sound waves or electric fields, enabling on-the-fly modifications to the focal length.

In a parallel development, researchers have introduced innovative optofluidic microlens arrays (MLAs) based on water-in-oil droplets featuring a controlled droplet diameter of 200 μm (Figure 13b) [83]. These MLAs can be seamlessly self-assembled in a microfluidic chip, and their refractive index can be precisely tuned by adjusting flow rates. This fine-tuning results in an impressive range of focal lengths, spanning from 550 to 5730 μm. Suspended within immiscible liquids, these liquid droplets not only control focal length but also offer high tunability, rapid response times, and a compact, lightweight nature. In contrast to electrowetting lenses, this technology is cost-effective and continually evolving. The MLAs, with their easy integration, find applications in diverse fields, including 3D imaging, stereoscopic analysis, biomedical sensing, and display technology [83,84].

### 4.4. Display and Information Tags

**Figure 14:**
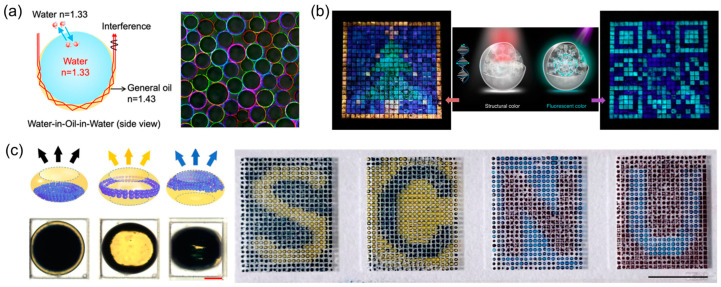
(**a**) Structural coloration in thin-shell emulsion droplets. Reflection optical micrographs of various structural colors in emulsion droplets with different sizes. Adapted from Ref. [92] with permission from American Chemical Society. (**b**) Geminate labels are formed by programming fluorescent CLC droplets, displaying QR code and Christmas tree under different conditions. Adapted from Ref. [95] under the terms of Creative Commons CC BY license. (**c**) A reflective display based on the electro-microfluidic assembly of particles. Adapted from Ref. [99] under the terms of Creative Commons CC BY license.

Droplets exhibit versatility as pixel units in visual presentations, evolving into active or passive displays, as well as information tags. One technological strategy entails utilizing droplets with structural color as photonic pigments, creating patterns for applications such as information display, encryption, and anti-counterfeiting [11,85,86,87]. This phenomenon originates from the interaction between the internal structures of droplets and light, providing advantages such as fade resistance, eco-friendliness, iridescence, high saturation, and intelligent stimulus response compared to methods involving chemical dyes. Diverse technological principles contribute to the formation of structural color in droplets, including the self-assembly of periodic structures using colloids and cholesteric LC within droplets and the creation of specific patterns through photonic cross-communications between droplets in an array (Figure 14a) [66,88,89,90,91]. 

Research has revealed that structural color can be achieved based on total internal reflection and interference at the microscale concave interface within double emulsion droplets with snowman-like and core–shell structures (Figure 14b) [92,93,94]. The adjustment of shell thickness and the eccentricity of core–shell structures enable the tuning of structural colors. In addition to structural color, another method involves incorporating dye into the same droplets to impart fluorescent color. By organizing these droplets in an ordered arrangement to form specific patterns, they can display different information under varying conditions, thereby enhancing information security (Figure 14c) [95,96,97]. 

Droplets can also serve as encapsulation units containing colored particles in their cavity. By using electric or magnetic fields to drive the arrangement of particles within the droplets, it becomes possible to adjust light transmittance, achieving information display and switching [98,99]. This approach to reflective displays has advantages such as energy efficiency, a wide field of view, and a relatively fast switching time (~0.14 s). Additionally, it enables the development of displays that can be curved or flexible. 

### 4.5. Other Applications

Exploring droplet-based sensor development constitutes a noteworthy research avenue. The standard technical strategy involves employing specially designed stimulus-responsive polymers to solidify periodic structures within the droplets. Consequently, the variations in the structural color of these droplets can be harnessed for detecting parameters like temperature, pressure, pH, specific components in solutions (e.g., metal ions, proteins, DNA), and even volatile gases, among others [100,101,102,103,104,105,106,107,108,109]. Such applications necessitate the utilization of smart materials, such as polymers or surfactants, to accomplish the mentioned functions. This characteristic renders this field a focal point in both chemistry and biology. 

## 5. Perspective and Conclusions

The paper provides a comprehensive overview of recent advancements in soft-matter droplet structure machining, utilizing techniques such as microfluidics, microfluidic 3D printing, and laser injection. Undoubtedly, microfluidics has been the most successful machining technique in microfabricating droplet structures. Substantial research efforts aim to enhance the capabilities of microfluidics, such as increasing droplet production through parallelizing more microchannels, cascading additional microchannels for multistep emulsification, or employing phase transition to assemble complex emulsion droplets. Furthermore, there are developments in chip-free methods and the introduction of 3D-printed fittings, eliminating the need for complex microfluidic chips and specialized expertise. However, owing to the unique physical properties of soft matter, microfluidics encounters challenges in fabricating small-sized and complex-structured droplets. More importantly, its top–down and direct-contact characteristics result in the processing of soft matter as ordinary liquids. The full potential to utilize the self-assembly of soft matter for droplet assembly remains to be exploited.

Microfluidic 3D printing and Laser injection share many similarities, employing a bottom–up strategy to assemble soft-matter droplets by injecting droplet cores into a host droplet using nozzle or laser irradiation. Additionally, both techniques require the assistance of microfluidic chips to accomplish certain functions. Microfluidic 3D printing is a kind of ink-based method and thus has relatively low precision. But it holds the potential to explore large-scale device-level applications owing to its high flexibility [73,110]. Laser injection technology introduces laser micro–nano-machining to the field of soft matter, offering relatively high precision and sub-micron machining scale. Importantly, it allows the machining of droplet inner architecture with unprecedented complexity alongside customized assemblies. With non-contact, in situ processing characteristics, it holds great promise to develop into a comprehensive fabrication platform for machining various types of soft matter. However, compared to microfluidics, both technologies have room for improvement in terms of maturity to gain widespread acceptance.

The potential of soft-matter microdroplets in optical and photonic applications is vast, offering adaptability, tunability, and seamless integration. Droplet structure machining technologies aim to tailor and control the physical functionality, optical properties, and dynamics of droplets by customizing their geometry and composition, making them a key driver for advancing frontier applications. Furthermore, as droplets inherently serve as encapsulation forms, each droplet can be developed into an independent functional unit, such as laser sources, lenses, optical waveguides, information pixels, etc. The formation of droplet arrays based on different functional droplets may provide opportunities for the development of multifunctional integrated optoelectronic chips.

Despite significant progress and great potential in this field, its development poses challenges. Due to the intricate nature of light and its transmission, applications in the optical and photonic domain demand higher precision, complexity, and stability in liquid control. The rapid development of laser droplets in photonics, along with cases like adjusting droplet morphology for liquid lenses by balancing interfacial tensions, further demonstrates this need. Drawing inspiration from broader fields of microfluidics, 3D printing and laser micro–nano-machining and combining them with the self-assembly properties of soft matter materials may be the key to the development of soft-matter microdroplet machining technology and application development.

## Data Availability

The data are available from the corresponding author upon reasonable request.
